# Arginine as an Enhancer in Rose Bengal Photosensitized Corneal Crosslinking

**DOI:** 10.1167/tvst.9.8.24

**Published:** 2020-07-14

**Authors:** Christian M. Wertheimer, Bryan Mendes, Qing Pei, Katharina Brandt, Irene E. Kochevar

**Affiliations:** 1 Wellman Center for Photomedicine, Massachusetts General Hospital, Boston, MA, USA; 2 Harvard Medical School, Boston, MA, USA; 3 Ludwig-Maximilians-Universität Munich, Ophthalmology, Munich, Bayern, Germany

**Keywords:** rose bengal, crosslinking, cornea, tensile strength, photochemistry, arginine

## Abstract

**Purpose:**

Oxygen-independent cornea crosslinking (CXL) using rose bengal (RB) and green light may have unique clinical applications. These studies were designed to gain insight into the arginine (arg)-enhanced anaerobic crosslinking process, to maximize crosslinking efficiency, and to test a clinically feasible method for oxygen-free CXL.

**Methods:**

Rabbit corneas were treated ex vivo using 1 mM RB and 532 nm light. RB photodecomposition, monitored by absorption spectrophotometry, was used to optimize arg concentration and to develop an irradiation and re-dying protocol. The minimal effective green light fluence was identified by linear tensile strength measurements. RB penetration into the stroma was determined by fluorescence microscopy. To favor the anaerobic pathway, a contact lens was used to minimize stromal oxygen level during irradiation. Stromal cell toxicity was evaluated by a terminal deoxynucleotidyl transferase-mediated deoxyuridine triphosphate nick-end labeling (TUNEL) assay.

**Results:**

RB photodecomposition reached 75% of its maximal effect at 200 mM arg and the optimal fluence increment was 32.7 J/cm^2^. The minimal effective fluence for cornea stiffening was 65.4 J/cm^2^. Placement of a contact lens promoted oxygen-independent cornea stiffening, similar to that obtained on isolated, oxygen-deprived cornea. RB penetration into the stroma with arg present was limited to ∼120 µm, about 25% deeper than without arg. Stromal cell toxicity was limited to the depth of RB and arg penetration.

**Conclusions:**

An oxygen-independent pathway in cornea for RB-CXL was characterized and optimized, including a possible clinical protocol for its use.

**Translational Relevance:**

Oxygen-independent RB-CXL is an efficient and effective process that can be developed further for unique clinical applications.

## Introduction

Photosensitized protein crosslinking using rose bengal (RB) and green light at 532 nm (RB CXL) has several demonstrated applications in the cornea, including stromal stiffening for treatment of ectatic diseases, such as keratoconus, photobonding of LASIK flaps to the corneal stroma, and sealing wounds and lacerations in preclinical studies.^1^^–^^5^ Recently, the first uses of RB photosensitization in a human patient's cornea was reported.^6^^–^^8^ The treatment halted a sight-threatening case of fusarium keratitis and, 16 months later, the cornea showed a persistent demarcation line in optical coherence tomography (OCT), a classical sign of corneal crosslinking.^7^

RB photosensitization differs from conventional riboflavin crosslinking in several ways. The RB molecules remain in a ∼100 µm layer of stroma near the epithelial surface rather than diffusing throughout as occurs for riboflavin.^9^^,^^10^ Thus, protein crosslinking and cornea stiffening is limited to this layer blocking any potential damage to the endothelium, as was shown in a model of ultra-thin rabbit corneas after photorefractive keratectomy in vivo.^11^ In addition, the RB application time and irradiation times are short (∼2 minutes and 10 minutes, respectively) and the loss of viable keratocytes is limited to the same depth as produced by removal of the epithelium alone.^10^

Our recent study indicated that RB crosslinking in the cornea is initiated by the two classical photosensitization pathways, depending on the treatment conditions.^9^ In an oxygen-independent pathway, after light absorption by RB, an electron is transferred from a donor molecule to the RB triplet excited state, producing radicals that lead to protein-protein crosslinks. In a second pathway, energy is transferred from the RB triplet excited state to oxygen generating a reactive oxygen species, singlet oxygen, that initiates crosslinking. In the absence of added agents, photo crosslinking required the presence of oxygen.^9^

A major finding of that study was that enhancers present during the irradiation make the crosslinking more efficient both in the presence and absence of oxygen. Especially important, given the reduced availability of oxygen in the stroma, we found that electron donor molecules, arginine (arg) in particular, promote RB crosslinking even in the absence of oxygen and that the crosslinking effect was the same as that in an oxygen environment without arg.

The goals of this study were to gain insight into the process of oxygen-independent photocrosslinking of cornea using RB, arg and green light, to maximize the efficiency of this process and to test a method for excluding oxygen that can be used in the clinic.

## Methods

### Tissue Preparation

Mature New Zealand white rabbit eyes freshly frozen were purchased from Pel-Freez Biologicals (Rogers, AR) and prepared as described previously.^9^ In brief, after removal of the epithelium, a corneoscleral disc was prepared and, for previously frozen eyes, placed in phosphate buffered saline (PBS; Fisher Bio Reagents, Fair Lawn, NJ) containing 15 to 20% w/v dextran (MW 450000—600000; Sigma, St. Louis, MO) until a pachymetry (Ganymed OCT; Thorlabs, Newton, NJ) of below 400 µm was reached. The epithelial surface of the stroma was stained with 1 mM RB (Sigma) or with RB plus arg (Sigma) both in dextran/PBS solutions.

### Light Source

A potassium titanyl phosphate crystal solid-state laser (Oculight OR; IRIDEX, Mountain View, CA) emitting 532 nm (green light) was used with a collimator to expand to a 1.2 cm diameter beam to deliver an irradiance of 0.22 W/cm^2^, as described previously.^9^

### Effect of Arg Concentration on RB Photobleaching

To determine the effect of different concentrations of arg on photobleaching of RB, 10 corneas were soaked in different arg concentrations from 0.1 to 800 mM in 20% w/v dextran in PBS for 30 minutes. Then, the epithelial side was dyed in 1 mM RB in PBS containing the same concentration of arg and the cornea was placed between two microscope glass slides equipped with a plastic spacer and surrounded by dextran/PBS solution to block contact with oxygen. Corneas were then re-dyed until showing an optical density (OD) of 0.9 to 1.4 (12.6–4.0% transmittance) at 532 nm and then irradiated with 8.2 J/cm^2^ green light. Absorption was measured (Cary 5000 Spectrophotometer; Agilent Technologies, Carpinteria, CA) before and after irradiation and the relative loss in absorbance at 560 nm was calculated. The arg concentrations producing 50 and 75% of the maximum effect (efficient concentration, EC_50_, EC_75_) were determined by fitting a sigmoidal dose response curve (4 parameter logarithmic regression) using Graph Pad Prism (GraphPad Software, La Jolla, CA).

### Effect of Green Light Fluence on RB Photobleaching

In order to maintain high efficiency for absorption of the green light, the fluence used should not greatly photobleach RB. The decrease in RB absorption as a function of light fluence was determined on corneas stained with 1 mM RB plus 200 mM arg and irradiated without oxygen, as described above. The absorption spectrum was measured after corneas were exposed to sequential fluences of 8.2 J/cm^2^.

### Penetration Depth of RB in Arg-Treated Corneas

Three corneas, with epithelium removed, were placed in 20% dextran solutions containing 200 mM arg to reach a thickness of below 400 µm and then stained with 1 mM RB plus 200 mM arg solution. Dying was continued until an absorption of ∼2.0 at the 560 nm peak was reached. The cornea was frozen in optimal cutting temperature solution (Tissue Tek, VWR, Rednor, PA) in a −80°C freezer and then kept on dry ice before cutting. Five µm sagittal cryo sections were cut using a kryo microkeratome at −20°C (CM 1510S; Leica, Wetzlar, Germany). Appropriate for RB, an excitation filter (510–560 nm) and an emission filter (590 nm) and a dichromatic mirrow (575 nm; built in G-2A filter; Nikon, Tokyo, Japan) were used to produce greyscale images using a fluorescence microscope (Eclipse TE2000; Nikon). Greyscale intensity plot profiles from the anterior to the posterior surfaces were taken at 10 locations per cornea using ImageJ 1.80 (National Institutes of Health, Bethesda, MD). All intensity profiles were averaged and plotted versus depth below the stromal epithelial surface.

### Linear Tensile Strength Testing

Different experimental groups were established (Table 1). In order to optimize and reduce the fluence applied, and due to more rapid photobleaching in arg-treated samples, lower fluences then previously used were applied (32.7 or 65.4 J/cm^2^). Non-irradiated samples served as controls. Corneas were either irradiated in air on the anterior chamber or, to provide oxygen-free conditions, the corneas were placed between two glass slides and surrounded by dextran solution. After irradiation, the tissue was kept in a moist chamber for at least 30 minutes before pachymetry was taken. The nasal temporal orientation of the eye was determined by the location of the two scleral vortice vessels and marked. At this location, a 5-mm wide nasal-temporal oriented corneal strip was prepared using fixed distance-separated razor blades. The exact width was measured by a digital caliper (Mitutoyo, Takatsu-ku, Japan). Force generated as a function of strain was determined using a linear tensile strength tester (eXpert 4000; Admet, Norwood, MA). Young's modulus was calculated between 0.1 and 5% strain at 0.1% intervals. All stress strain curves were fitted using a fifth order polynomial in Matlab R2018a (Math Works, Natick, MA). The slope was calculated by finding the second derivative to give the Young's Modulus at 0.1% strain intervals.

**Table 1.** Summary of Cornea Thickness and Group Size for all Experimental Groups

Group	Pachymetry, µm	Sample Size, *n*
Untreated control	309 ± 32	21
Arg	375 ± 33	19
RB, arg, no irradiation	400 ± 33	10
RB, arg, 32.7 J/cm^2^, no O_2_	399 ± 36	9
RB, arg, 65.4 J/cm^2^, no O_2_	389 ± 31	7
RB, 65.4 J/cm^2^, no O_2_	318 ± 21	10
RB, arg, 65.4 J/cm^2^, contact lens	362 ± 28	14
RB, arg, 65.4 J/cm^2^, O_2_	323 ± 21	9
RB, 65.4 J/cm^2^, O_2_	311 ± 12	11
RB, arg, no irradiation, contact lens	367 ± 23	6

Cornea thickness was measured by pachymetry.

### Potential Clinical Method for Oxygen-Independent RB CXL

Arginine enhances RB CXL only when oxygen is not available.^9^ A method was developed to allow irradiation in a low oxygen setting in the cornea, which might be used in a later clinical application. This was achieved by placing a corneoscleral disk onto the artificial anterior segment to guarantee natural curvature. After dehydration in 200 mM arg in 20% w/v dextran, the cornea was then dyed, as described above, with 200 mM arg in 1 mM RB in PBS. To block oxygen diffusion into the cornea during irradiation, a contact lens (HP Sphere; Alden Optical, Lancaster, NY) with curvature and diameter equal to the rabbit anterior corneal surface was placed on the cornea. Oxygen-free conditions were confirmed by observation of the shift of the absorption maximum toward 510 nm that accompanies anaerobic photobleaching of RB in the presence of arg.^12^ Corneas were either irradiated with 32.7 or 65.4 J/cm^2^. This was followed by linear tensile strength testing, as described above. Corneas without arg and contact lens served as controls.

### Evaluation of Toxicity to Stromal Cells

Possible toxicity to keratocytes from arg-enhanced crosslinking treatment was evaluated by transferase-mediated deoxyuridine triphosphate nick-end labeling (TUNEL) staining for apoptotic cells. For this purpose, fresh mature New Zealand white rabbit eyes were used within 24 hours of death. The whole globes with intact epithelium were disinfected in 1% w/v povidone iodine solution in PBS for 5 minutes (Medline, Northfield, IL). All further experimental steps were conducted in a sterile setting. The epithelium was removed with a hockey knife (Katena, Parsippany, NJ) and the cornea was stained with 1 mM of RB in PBS and, depending on the experimental procedure, 200 mM Arg. After dying, the eye was either irradiated with green light in room air or placed in a chamber with a transparent window for irradiation and a port for delivering N_2_ to wash out air and thus oxygen. The total fluence was 82 J/cm^2^. After irradiation, a corneoscleral disc was prepared using a trepan and keratoplasty scissors (Katena, Parsippany, NJ). Afterward, a suture was placed through the scleral rim of the corneoscleral disk to suspend it in cell culture medium in an upright 75 mL cell culture bottle. To allow a cellular response, the tissue was incubated at 37°C and 5% CO_2_ for 72 hours in medium (MEM Earls; Sigma) supplemented with 2% fetal calf serum, 100 units penicillin/mL, 100 µl streptomycin/ml and 1 ug/mL amphotericin B (all from Thermo-Fisher Scientific, Waltham, MA). After cultivation, the tissue was fixed using 10% buffered formalin phosphate for 48 hours and embedded in paraffin. Five µm sections were stained with hematoxylin and eosin, then TUNEL staining was performed using a commercial assay following the manufacturer's instructions (DeadEnd, Fluorometric TUNEL System; Promega, Fitchburg, WI). Representative images of the samples were saved using a fully automated slide scanner (Nanozoomer; Hamamatsu Photonics, Hamamatsu City, Japan). The depth to which TUNEL-positive cells appeared was analyzed on a central cornea section at a minimum of three different points in the irradiated area on each cornea.

### Statistical Analysis

Statistical comparison between experimental groups was carried out using an ANOVA with an least significant difference (LSD) post hoc test. For all analyses, *P* < 0.05 was considered statistically significant. All graphs were plotted in Prism 8 (GraphPad Software) with the mean displayed and the standard deviation as an error bar. All statistical analyses were performed by SPSS 24.0 (IBM, Armonk, NY) and all calculations were done either by Excel 365 (Microsoft; Albuquerque, NM) or MATLAB 9.3 (MathWorks, Natick, MA).

## Results

### Effects of Arginine

Our previous results indicated that in the absence of oxygen, RB CXL occurred by a mechanism involving transfer of an electron from arg to the triplet excited state of RB.^9^ To learn about other aspects of the CXL process, we investigated the influence of arg on penetration of RB into the stroma and the arg concentration dependence on RB photochemistry in cornea. Corneas were treated with 1 mM RB and 200 mM arg, fixed, and examined by fluorescence confocal microscopy. A layer of RB was close to the anterior surface. However, the average depth of penetration (10% of the highest dye intensity) was 119 µm in a ∼500 µm thick cornea (Supplementary Fig. S1), deeper than that observed for RB alone (∼87 µm).^9^

**Figure 1.**
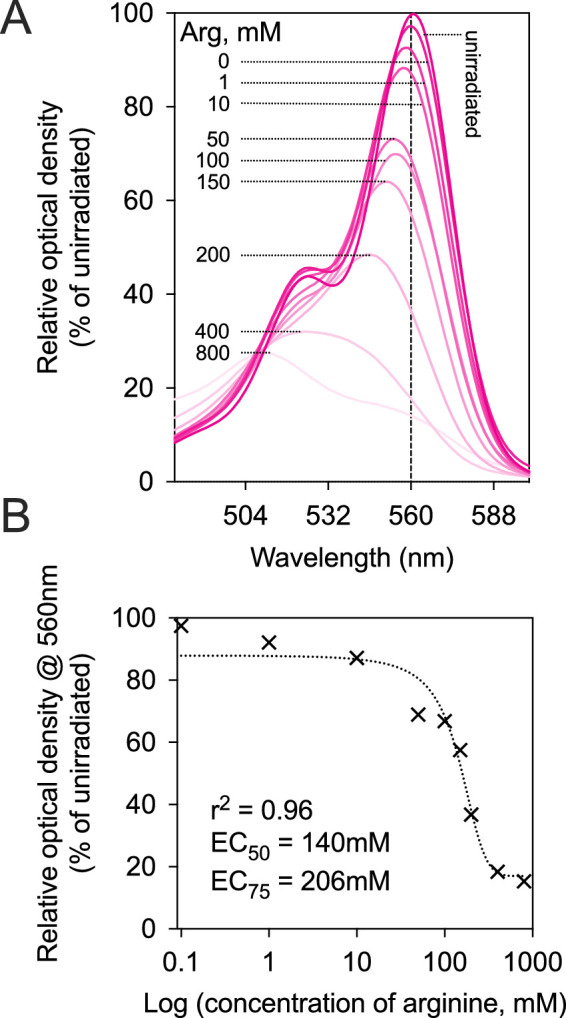
Influence of arg concentration on photobleaching of Rose Bengal in cornea. After staining with 1 mM RB solutions and dehydration with dextran solution, both containing varying concentrations of arg, corneas were irradiated with 8.2 J/cm^2^ green light in the absence of oxygen. (**A**) RB absorption spectra before and after irradiation. (**B**) The RB absorption at 560 nm after 8.2 J/cm^2^ irradiation versus arg concentration.

To estimate the optimal arg concentration for anaerobic RB CXL, we measured RB photobleaching because our previous study demonstrated that the increase in cornea tensile strength paralleled RB photobleaching.^9^ Both, the rose Bengal solutions (1 mM) and the dextran solution for dehydration containing arg concentrations between 0.1 and 800 mM were used together to stain corneas. Absorption spectra of the corneas were measured before and after irradiation with 8.2 J/cm^2^. Photobleaching increased significantly with increasing arg concentration (Fig. 1A). The absorbance at 560 nm was plotted versus the log arg concentration (Fig. 1B). A sigmoidal dose response curve could be fitted where the response was defined the loss of absorption at 560 nm peak (r^2^ = 0.96). The arg concentration that produced 75% of the response between the minimum and maximum effect (EC_75_) was determined from the sigmoidal dose response curve fit to be 206 mM. Surprisingly, this value is nearly identical to the arg concentration (200 mM) that was used in the previous study, and thus was chosen for use in further experiments.

### Optimizing Green Light Irradiation Treatment

**Figure 2.**
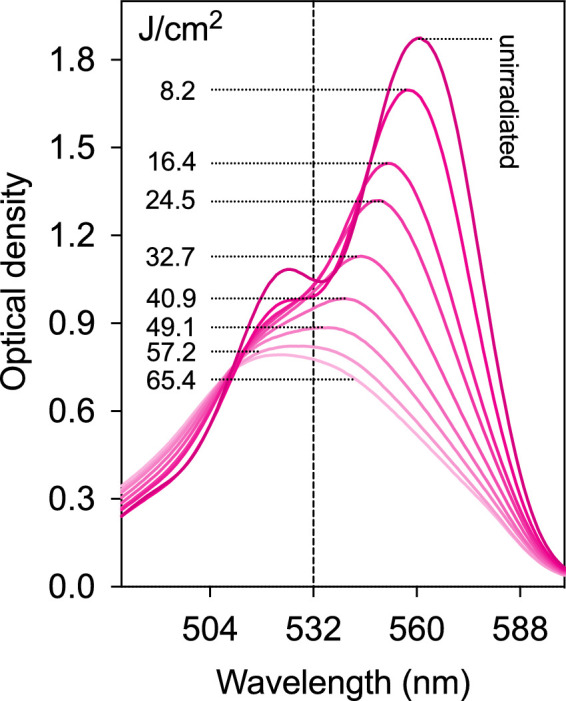
Absorption spectrum of a cornea treated with 1 mM RB and 200 mM arg before and after irradiation with sequential 8.2 J/cm^2^ fluences of green light in the absence of oxygen.

For clinical applications, a lower fluence (J/cm^2^) to achieve the maximum increase in tensile strength has the advantages of a shorter irradiation time and potentially a lower possibility of adverse side effects. We investigated whether it was possible to produce effective RB CXL at lower fluences than used in our prior study (200 J/cm^2^).^9^ First, for highly efficient photo-crosslinking, it was important to maximize the amount of light absorbed throughout the irradiation period. After dying the cornea until an optical density of 1.0 at 532 nm, the cornea absorbs about 90% and transmits only about 10% of the incident green light. However, irradiation partially destroys (photobleaches) some of the RB and thus decreases the efficiency of light absorption, and consequently photo-crosslinking. The RB-stained cornea was irradiated with sequentially with 8.2 J/cm^2^ in order to determine the maximum fluence that would maintain the transmittance at 532 nm below 10% (absorbance = 1). As shown in Figure 2, after irradiation fluences greater than 32.7 J/cm^2^ the absorption fell below 1. However, for tensile strength measurements a single 32.7 J/cm^2^ irradiation was not effective as shown below (Table 2 and Supplementary Fig. S2). Consequently, after 32.7 J/cm^2^ was applied, the cornea was re-dyed with RB/Arg before a subsequent exposure to 32.7 J/cm^2^ for stiffness measurements.

**Table 2.** Comparisons of Young's Modulus of Control and Treated Corneas and Results of Statistical Testing

Comparison		
Group 1	Group 2	Range of Percent Strain for which *P* < 0.05 between Groups for Young's modulus
Control	RB, arg, 32.7 J/cm^2^, no O_2_	None
Arg	RB, arg, 32.7 J/cm^2^, no O_2_	None
RB, arg, no irradiation	RB, arg, 32.7 J/cm^2^, no O_2_	None
RB, 65.4 J/cm^2^, no O_2_	RB, arg, 32.7 J/cm^2^, no O_2_	None
Control	RB, arg, 65.4 J/cm^2^, no O_2_	0.3–5
Arg	RB, arg, 65.4 J/cm^2^, no O_2_	0.3–5
RB, arg, no irradiation	RB, arg, 65.4 J/cm^2^, no O_2_	0.4–5
RB, 65.4 J/cm^2^, no O_2_	RB, arg, 65.4 J/cm^2^, no O_2_	0.8–5
RB, arg, 32.7 J/cm^2^, no O_2_	RB, arg, 65.4 J/cm^2^, no O_2_	1–3.7

**Figure 3.**
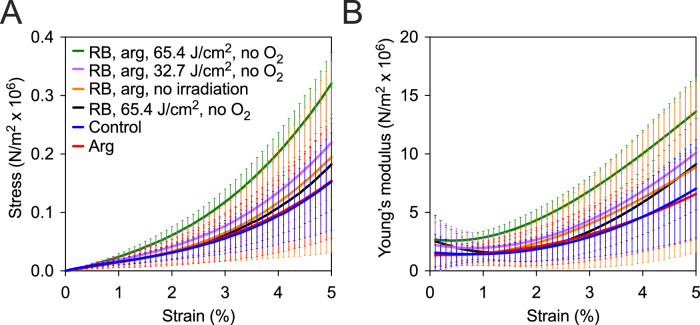
Effect of varying the fluence of green light on the tensile strength increase induced by RB photosensitized cornea crosslinking. All corneas were irradiated in the absence of oxygen except the three controls (no treatment, arg only, and RB, arg) were not irradiated and (**A**) stress-strain curves from tensile strength testing and (**B**) Young's modulus versus percent strain.

To determine whether fluences lower than 200 J/cm^2^ were effective for anaerobic RB CXL, corneas were dehydrated in dextran solution and stained with 1 mM RB and 200 mM arg, then were treated with either 32.7 or 65.4 J/cm^2^ in the absence of oxygen. The tensile strength of these corneas was then compared to these three unirradiated controls (no treatment or 200 mM arg only or 1 mM RB plus 200 mM arg). Figure 3A shows the stress strain curves up to 5% strain for all treatment conditions and Figure 3B shows Young's modulus over the same range of percent (for exact values see Supplementary Figure S2) strain. A significant increase in Young's modulus was observed across almost the entire range of percent strain between each of the 4 controls and corneas that were irradiated with 65.4 J/cm^2^ arg and no oxygen (see Table 2). However, corneas exposed to the lower fluence, 32.7 J/cm^2^, were not significantly different compared to any of the controls. The Young's modulus differed significantly between samples irradiated with 32.7 and 65.4 J/cm^2^. Corneas that were not treated with arg, but irradiated with 65.4 J/cm^2^ in oxygen free condition were significantly different compared to those with arg and irradiation.

### Potential Clinical Approach for Oxygen-Independent Crosslinking

**Figure 4.**
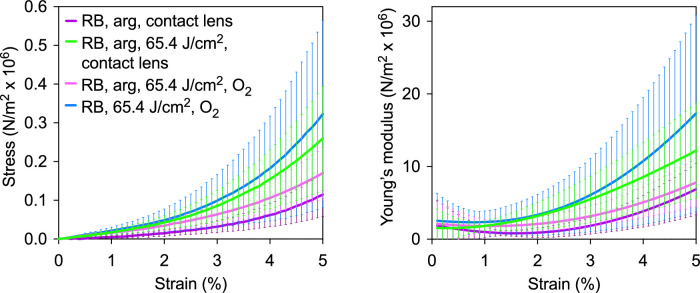
Effect on the cornea tensile strength of the presence of a contact lens during irradiation in the presence of RB and arg, but absence of oxygen. (**A**) Stress-strain curves from tensile strength measurements and (**B**) Young's modulus versus percent strain.

Our laboratory approach to exclude oxygen involving irradiating the cornea between glass slides for RB CXL is clearly not feasible for clinical applications. Consequently, we tested an approach in which a standard, easily available gas permeable contact lens was applied to the cornea after staining with the RB/arg solution and remains in place during the irradiation. We hypothesized that the permeability through the contact lens is too low to counteract the rapid use of oxygen occurring during crosslinking.^13^ Irradiating RB/arg-stained corneas with the contact lens in place significantly increased Young's modulus compared to control corneas treated the same way without irradiation (Fig. 4, Table 3 and Supplementary Fig. S2). This contrasts with irradiation of RB/arg-stained corneas without the contact lens (i.e. oxygen is present), which did not differ from the same control and demonstrates that the contact lens effectively blocked oxygen diffusion into the cornea. The tensile strength attained after irradiation with the contact lens was similar to that observed after irradiation with RB but not arg in the presence of oxygen.

**Table 3.** Comparisons of the Increase in Cornea Tensile Moduli and Results of Statistical Testing

Comparison		
Group 1	Group 2	Range of Percent Strain for which *P* < 0.05 between Groups for Young's modulus
RB, arg, no irradiation, contact lens (control)	RB, arg, 65.4 J/cm^2^, contact lens	1.2–3.4
RB, arg, no irradiation, contact lens (control)	RB, 65.4 J/cm^2^, O_2_	0.9–5
RB, arg, no irradiation, contact lens (control)	RB, arg, 65.4 J/cm^2^, O_2_	None
RB, 65.4 J/cm^2^, O_2_	RB, arg, 65.4 J/cm^2^, contact lens	3.3–5

### Evaluation of Phototoxicity to Stromal Cells

**Figure 5.**
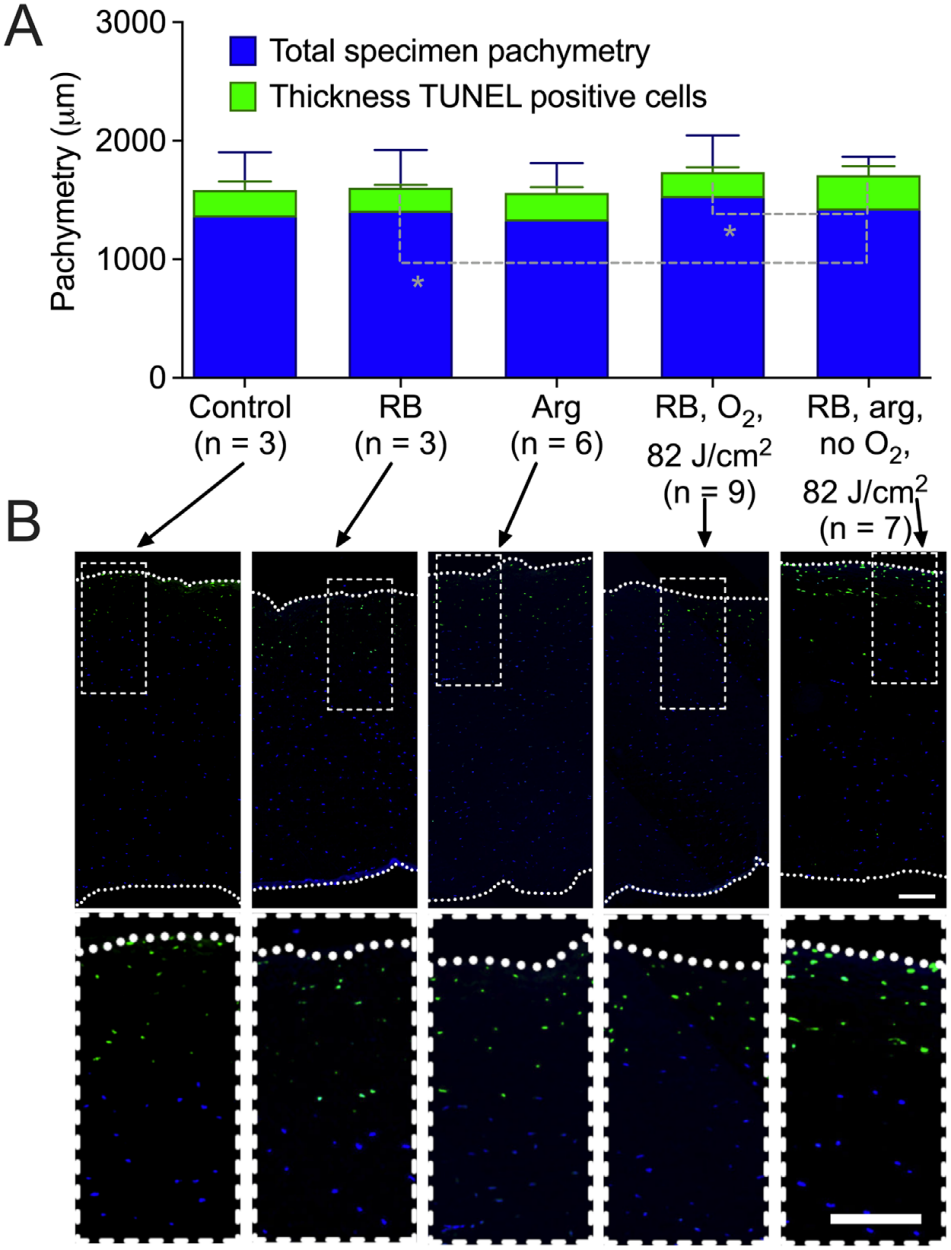
The blue-colored columns depict the absolute corneal thickness as measured from the microcopy specimens, whereas the green colored columns show the absolute thickness of TUNEL-positive cells, which were located below the previously removed epithelium in the anterior stroma (part A). Part B shows representative examples of stroma sections showing apoptotic cells. White dashed lines mark the area of enlargement from the images in the top row to the images in the bottom row. Non-enlarged top row images have a 5 times magnification, the bottom row images are digitally enlarged by a further 2.5 times from the original image. White dotted lines represent the Bowman layer (top) and the endothelial surface (bottom; scale bar = 200 µm).

Because RB-CXL involves reactive radicals that potentially can damage stromal cells, cell death was evaluated. Corneas from fresh ex vivo, but not previously frozen, rabbit eyes were stained with RB and/or arg, then irradiated, excised, and cultured to allow a cellular response. The three controls were untreated, stained with RB only, or treated with arg only. As expected during corneal cell culture, the corneas developed stromal edema causing an approximately four times thickness increase after preparation as a microscopy specimen. The absolute thickness of the corneal microscopy specimens did not vary significantly in any experimental group (*P* > 0.23 in all comparisons). In all groups, there was a layer of dead (TUNEL positive) cells always located in the anterior part of the cornea below the previously removed epithelium. TUNEL staining was observed even in the nontreated and RB-only and arg only controls to a depth of about 15 ± 1.5%, 14 ± 1.6%, and 16 ± 3.5% of the total stroma thickness, respectively, which was not statistically significant when the absolute depth was compared (*P* > 0.49 in all comparisons; Fig. 5). Experimentally, the depth of TUNEL-positive cells in the corneas was also about the same in the RB only irradiation in air (13 ± 1.6% of the whole thickness), which was confirmed by the absence of a statistically significant difference (*P* > 0.52 in all comparisons to the controls). Interestingly, the depth of TUNEL-positive cells increased slightly after irradiation in the absence of oxygen with arg present, to 18 ± 4.6%. This was significantly higher than in the RB and air irradiation group (*P* = 0.009) and the RB only control (*P* = 0.03) and might be attributed to the larger penetration depth, as we have shown above when arginine is present.

## Discussion

This study identified similarities and differences between oxygen-independent (arg-mediated) and oxygen-dependent mechanisms for RB CXL, established optimal conditions for oxygen independent crosslinking, and demonstrated a clinically applicable method for oxygen-independent RB-CXL. The presence of arg allows deeper, but still limited, penetration of RB into the stroma. Approximately the same minimal fluences of green light are required for oxygen-independent RB CXL, as previously shown for the oxygen-dependent process. Stromal cell death was detected only close to the anterior surface, extending to the same depth as RB penetration, similar to that found for oxygen-dependent crosslinking and de-epithelialization alone. Using a gas impermeable contact lens, a practical clinical approach for oxygen-independent cornea stiffening was demonstrated.

The minimal irradiation fluence that significantly stiffened the cornea with RB CXL in the absence of oxygen was shown to be about 65 J/cm^2^, substantially lower than the 200 J/cm^2^ used in our initial study on arg-enhanced crosslinking.^9^ A three-fold decrease in the time required for a CXL treatment is important for development of useful clinical applications. The total treatment time for RB/arg application and reapplication and irradiation is about 13 minutes. Using a higher irradiance, if safe, would shorten the optimal treatment time further. The irradiance used, 0.22 W/cm^2^, is slightly lower than that used in a previous study for RB-initiated crosslinking rabbit cornea where retinal damage was not observed.^2^ Interestingly, the minimal fluence (65 J/cm^2^) is somewhat lower than the minimal fluence reported previously for oxygen dependent CXL (100 J/cm^2^).^9^^,^^10^ Oxygen-dependent CXL was not effective at 50 J/cm^2^ (unpublished results). Oxygen-dependent and oxygen-independent pathways for CXL involve different mechanisms, energy transfer, and electron transfer, respectively.^12^ The oxygen independent pathway involves transfer of an electron from an appropriate donor molecule, such as arginine, to the excited triplet state of RB to form the short-lived anion radical of RB and cation radical of arginine.^9^^,^^14^^,^^15^ The reactive species formed by the two mechanisms initiate formation of different protein-protein crosslinks and also have different efficiencies for crosslinking soluble proteins.^16^ The reason for the similar minimal effective fluences for aerobic and anaerobic RB CXL is not clear and requires further investigation.

Arg increased the penetration of RB into the stroma by about 25% (Supplementary Fig. S1, Supplementary Fig. S2), although the dye was still limited to the uppermost ∼120 µm and thus could not damage the endothelium during RB CXL. The limited penetration of RB into stroma in the absence of arg likely results from its strong association with collagen^12^ and the fact that it is aggregated at the high concentration applied (1 mM). RB remains mainly aggregated in the cornea, judging from the absorption spectrum, and large aggregates may not be able to diffuse through the stroma. Arg, which can diffuse through mouse cornea,^17^ could affect either or both of these factors, resulting in deeper penetration into the stroma. The RB anion, with two negative charges, associates strongly with positively charged amino acids on collagen, as shown in our previous study.^12^ Positively charged arg molecules may interfere with this association, thus allowing deeper diffusion of RB. Alternatively, interaction of arg with the aggregates might partially cause disruption, allowing deeper penetration of smaller aggregates or monomers.

We tested whether RB CXL with arg would still be effective when the cornea was intentionally deprived of oxygen using a method more relevant to clinical applications than our laboratory approach. The results demonstrate that arg effectively enhances RB CXL in corneas owing to a decreased rate of oxygen diffusion provided by a contact lens compared to unirradiated controls. In our experiments, the level of stiffening as measured by tensile strength testing was similar to that found for oxygen-dependent RB CXL. Thus, when an anaerobic environment is required, or a tissue has a low O_2_ level, this result indicates that the arg-enhanced oxygen-dependent CXL can be produced in situ. In fact, the singlet oxygen-initiated crosslinking photosensitized by rose Bengal and the oxygen independent crosslinking (initiated by electron transfer from arginine to the rose Bengal excited triplet state) are complementary. If the oxygen level is sufficient in the tissue, the singlet oxygen mechanism prevails; if insufficient oxygen is present, the oxygen-independent mechanism occurs. Both mechanisms increased the tensile strength to the same extent, as shown in our previous study.^9^ The photobleaching results indicated that rose Bengal in the presence of both arginine and oxygen favors the singlet oxygen pathway and deiodination does not occur as no blue shift in the spectrum can be observed. These results indicate that including arginine allows use of as much of the available oxygen as possible (to produce singlet oxygen crosslinking) and then, when oxygen is depleted or if the tissue has very low oxygen, allows oxygen independent crosslinking.^9^

Although oxygen-dependent CXL with riboflavin and UVA is currently used clinically, there are potential crosslinking applications in which the oxygen level is too low. An example is the linking of the capsular bag to intraocular lens (IOL) haptics using RB CXL technology, which required an external supply of oxygen for efficient crosslinking.^5^ An oxygen-independent method may make this application feasible. Other examples are vessel bypasses,^18^ tendons, and cartilage,^10^ or acellular nerve fiber grafts.^19^ All of those surgical tissue grafts are devoid of their natural vessel supply and have low oxygen availability. The same oxygen deprived conditions also apply to any deeper lamellar approaches within the cornea, including corneal incisions^9^ and laser-assisted in situ keratomileusis (LASIK) flaps.^4^ Because in a clinical setting the cornea will likely never be fully deprived of oxygen, some of the crosslinking must be attributed to the singlet oxygen pathway. However, as described previously, the oxygen dependent (singlet oxygen) and oxygen-independent pathways produce are complementary and produce equivalent crosslinking.^9^ Including arginine allows efficient use of incident light by promoting crosslinking after the available oxygen is depleted.

Toxicity in the cornea is an unwanted complication that might lead to cloudiness, neovascularization, scarring, and loss of vision. Riboflavin and UV crosslinking has been described to cause rare but serious side effects, like postoperative infective ulcer, persistent corneal haze, full keratocyte apoptosis in the entire crosslinked stroma, endothelial damage, peripheral sterile infiltrates, and herpes simplex reactivation.^20^ The effect and toxicity of RB in corneal crosslinking has also been well established in preclinical studies,^11^^,^^21^ and experience with RB plus green light is being accumulated in human studies.^6^^–^^8^

After crosslinking, it causes cell death in the cornea comparable to the approved and clinically widely used riboflavin and UV corneal crosslinking.^5^ Yet, there is no data on arginine and RB in combination. Arginine appears to be a safe choice for potential clinical applications. As an ubiquitous amino acid, it is well characterized and present in virtually all food,^22^ tissue, and is part of many US Food and Drug Administration (FDA) and European Medicines Agency (EMA) approved formulations. It has been used in intravenous formulations in humans in high concentrations^23^ and has been applied as an intravitreal injection to minipigs for retinal vein occlusions.^24^ Importantly, arginine has been used in concentrations comparable to those in this study as eye drops (2%) in mice and diffused through the cornea to reach the lens.^17^ Effects of RB CXL with arg on stromal cells was limited to the region closest to the anterior surface below the Bowman membrane, similar to that found for aerobic RB CXL, and substantially less deep than produced by riboflavin CXL.^5^ As previously reported, after oxygen-dependent RB CXL, cell death occurred only to the same depth as found after de-epithelialization alone (no treatment control; see Fig. 5), which is about the same depth as RB penetration into the stroma.^5^ After anaerobic RB CXL with arg, TUNEL-labeled cells were found at a slightly but statistically significant increased depth from the anterior surface, which correlates well with the increased penetration depth of RB in arg pretreated corneas. Further studies have to determine whether this increase has any consequences in a clinical setting, especially when compared to the results observed in riboflavin crosslinking.

Another possible future direction for research would be to use a different dye. It is important to choose a dye with a high quantum yield for production of singlet oxygen as well as having an excited triplet state that efficiently accepts electrons from arginine or another electron donor. Other xanthene dyes, such as erythrosin B, may be good candidates.^25^ One advantage of Rose Bengal is its long history of use in human corneal tissue, several demonstrated light-initiated corneal applications in preclinical studies, and recent clinical studies in human patient's cornea.^6^^–^^8^

In summary, RB-photosensitized crosslinking in the absence of oxygen can be enhanced by electron donors to produce the same level of stiffness exhibited in the presence of oxygen. A systematic evaluation of treatment parameters led to an optimization and a greater photochemical understanding of the described method. The reduced cell viability after the procedure seems acceptable and in concordance with the clinically used riboflavin crosslinking. We suggest that these findings will fuel several additional potential clinical applications.

## Supplementary Material

Supplement 1

Supplement 2
