# Supplementary material for: Arginine as an Enhancer in Rose Bengal Photosensitized Corneal Crosslinking
Source: Transl Vis Sci Technol. 2020 Jul 14;9(8):24. doi: 10.1167/tvst.9.8.24 (PMC7422776; doi:10.1167/tvst.9.8.24)
Supplement: Supplement 1 [file tvst-9-8-24_s001.pdf]

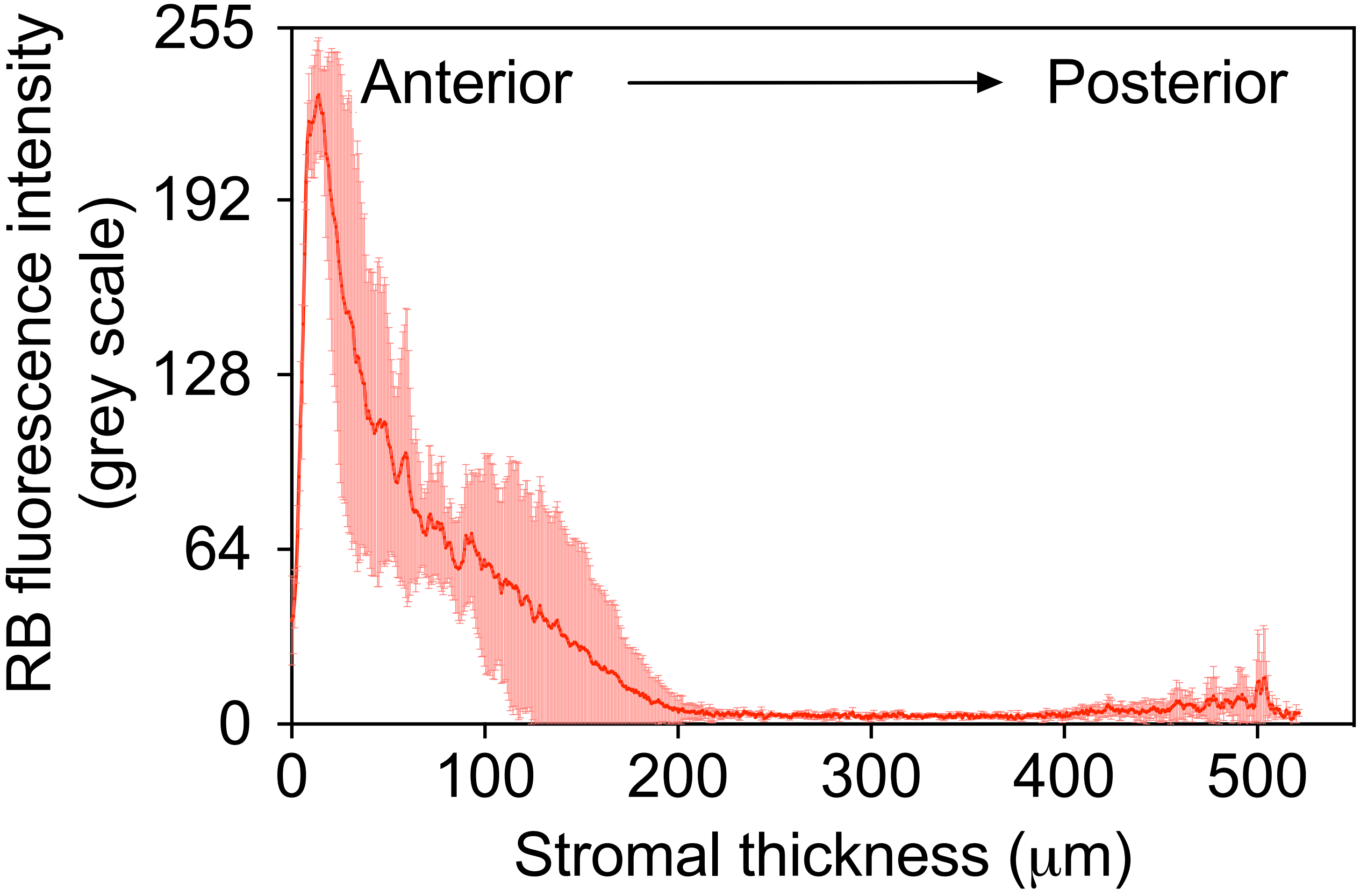

Figure S1: Fluorescence of RB in a sagittal cryosections of RB-stained corneas were photographed under a fluorescence microscope and greyscale profiles of the intensity were taken at 10 locations on each of three corneas, averaged and shown as a solid line with ± 1 SD. The fluorescence decreases to 10% of its original value at 119 μm below the.
