# Supplementary material for: Arginine as an Enhancer in Rose Bengal Photosensitized Corneal Crosslinking
Source: Transl Vis Sci Technol. 2020 Jul 14;9(8):24. doi: 10.1167/tvst.9.8.24 (PMC7422776; doi:10.1167/tvst.9.8.24)
Supplement: Supplement 2 [file tvst-9-8-24_s002.pdf]

Groups	p-Value	Groups	p-Value
Control, no irradiation **vs.** 65.4 J/cm^2, RB, arg, no O_2	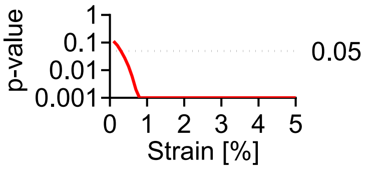	65.4J/cm^2, RB, no O_2 **vs.** 32.7J/cm^2, RB, arg, no O_2	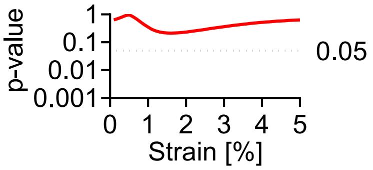
Control, arg, no irradiation **vs.** 65.4 J/cm^2, RB, arg, no O_2	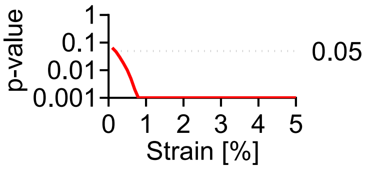	Control, no irradiation **vs.** Control, arg, no irradiation	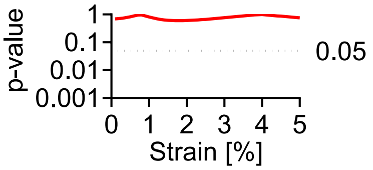
Control, RB, arg, no irradiation **vs.** 65.4 J/cm^2, RB, arg, no O_2	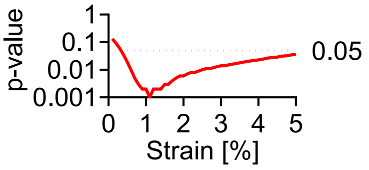	Control, no irradiation **vs.** Control, RB, arg, no irradiation	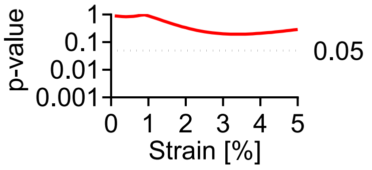
65.4 J/cm^2, RB, no O_2 **vs.** 65.4J/cm^2, RB, arg, no O_2	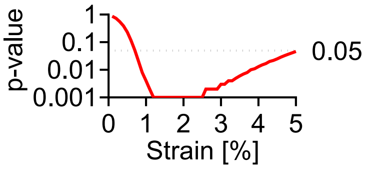	65.4J/cm^2, RB, arg, O_2 **vs.** 65.4J/cm^2, RB, arg, contact lens	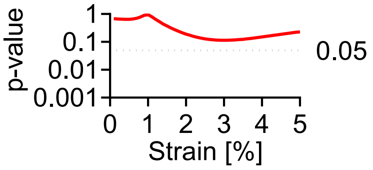
32.7 J/cm^2, RB, arg, no O_2 **vs.** 65.4 J/cm^2, RB, arg, no O_2	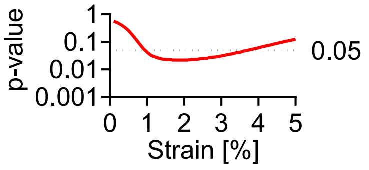	65.4J/cm^2, RB, O_2 **vs.** 65.4J/cm^2, RB, arg, contact lens	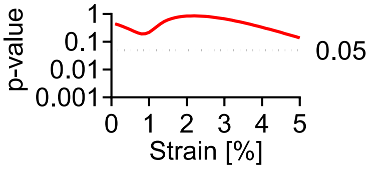
Control, no irradiation **vs.** 32.7J/cm^2, RB, arg, no O_2	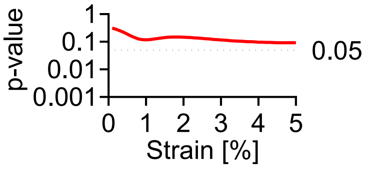	65.4J/cm^2, RB, O_2 **vs.** 65.4J/cm^2, RB, arg, O_2	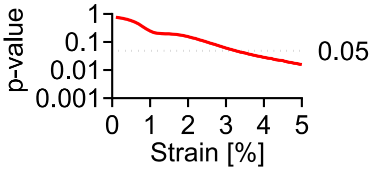
Control, arg, no irradiation **vs.** 32.7J/cm^2, RB, arg, no O_2	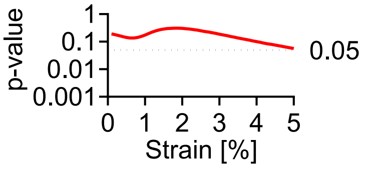	RB, arg, no O_2, contact lens control **vs.** 65.4J/cm^2, RB, arg, contact lens	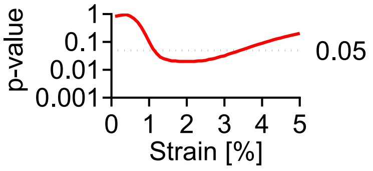
Control, RB, arg, no irradiation **vs.** 32.7J/cm^2, RB, arg, no O_2	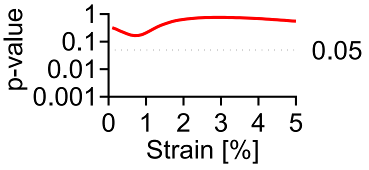		

Figure S2: The actual p-values are shown for scientifically meaningful comparisons in the tensile strength testing experiments (ANOVA, LSD post hoc test).
